# Is systems biology a promising approach to resolve controversies in cancer research?

**DOI:** 10.1186/1475-2867-12-12

**Published:** 2012-03-26

**Authors:** Ana M Soto, Carlos Sonnenschein

**Affiliations:** 1 Department of Anatomy and Cellular Biology, Tufts University School of Medicine, 136 Harrison Ave., Boston, MA 02111, USA

**Keywords:** Carcinogenesis, Default state, Systems biology, Proliferation, Oncogenes

## Abstract

At the beginning of the 21st century cancer research has reached an impasse similar to that experienced in developmental biology in the first decades of the 20th century when conflicting results and interpretations co-existed for a long time until these differences were resolved and contradictions were eliminated. In cancer research, instead of this healthy "weeding-out" process, there have been attempts to reach a premature synthesis, while no hypothesis is being rejected. Systems Biology could help cancer research to overcome this stalemate by resolving contradictions and identifying spurious data. First, *in silico *experiments should allow cancer researchers to be bold and *a priori *reject sets of data and hypotheses in order to gain a deeper understanding of how each dataset and each hypothesis contributes to the overall picture. In turn, this process should generate novel hypotheses and rules, which could be explored using these *in silico *approaches. These activities are significantly less costly and much faster than "wet-experiments". Consequently, Systems Biology could be advantageously used both as a heuristic tool to guide "wet-experiments" and to refine hypotheses and test predictions.

## 

The history of science shows that knowledge is acquired through the competition among alternative theories. Only after these theories are thoroughly explored, its components tested and validated, opportunity for a synthesis may arise. At such a time, contradictions may be resolved and both spurious "facts" and wrong premises can be recognized and dismissed. A misguided, premature synthesis may, instead, lead to an "anything goes" attitude where any given interpretation and its opposite happily coexist, incoherence is accepted as being the inexorable consequence of dealing with complexity. In such an instance, everything is explained because if results do not fit one theory, they may fit its opposite or an *ad hoc *alternative one. As room is made to reconcile every improper fit, there is no chance to rule out any of them. This attitude subverts the objectives of science as described by Ayala, namely: "First, science seeks to organize knowledge in a systematic way by exhibiting patterns of relations among statements concerning facts which may not appear obviously as mutually related... It is the second distinctive characteristic of science that it strives to provide explanations of why the observed events do in fact occur. Science attempts to discover and to formulate the conditions under which the observed facts and their mutual relationships exist. Thirdly, the explanatory hypotheses provided by science must be genuinely testable, and therefore subject to the possibility of rejection [1]."

An example of such a premature synthesis of opposing hypotheses in the biological sciences took place about a century ago. Jane Maienschein [2] quoted embryologist Herbert S. Jennings who when assessing the state of embryology recalled that different embryologists did similar experiments and arrived at quite different conclusions. "All the conflicting reports were correct. The situation was that of the Gilbertian comic opera chorus, 'For you are right, and I am right, and he is right and all is right'". Maienschein's analysis implied that epistemology does matter, and that competition between or among hypotheses is indeed fruitful.

The experiments done by Wilhelm Roux and Hans Driesch on the developmental potential of blastomeres probably are the best illustration of the above-referred situation. Roux believed that differentiation was driven by a mosaic pattern of development, in which the generation of phenotypic diversity resulted from unequal segregation of the genetic material among blastomeres and their progeny, as was previously proposed by August Weismann. Roux's experimental approach to explore this process was to destroy one of the two blastomeres resulting from the first cleavage, and the result of this intervention was a "half-embryo" as he predicted. Driesch expected to find similar results, but his experimental approach instead entailed the separation of the blastomeres. The resulting embryos were normal but smaller. He discovered "regulative" development, whereby a single blastomere was able to generate a whole embryo. He abandoned Roux's hypothesis in light of these results. Roux stuck to his interpretation on the grounds that his theory explained many facts, and to justify Driesch's results he proposed the *ad hoc *explanation that a "reserve germ plasm" existed in the blastomeres. Parenthetically, Roux used frog and Driesch used sea urchin embryos; both undergo regulative development as first shown by Driesch. The impasse was resolved when embryologists concluded that, during the first and second cleavages, embryonic development could undergo either a regulated or a mosaic pattern of development, the first giving occasionally identical twins; however, any given species undergoes only one of the two. This historical example shows that it is indeed difficult, if not impossible, to advance knowledge without a clear epistemology to guide experimental design, data gathering, and their interpretation into evidence for or against a given hypothesis.

Parenthetically, it was the American embryologist Edmund B. Wilson who even-mindedly proposed that regulative and mosaic development were the extremes of a gradient [3]. This perspective is now prevalent because at specific times during development, a given cell may behave as a mosaic, its fate being specified by its history, and at the next stage its fate is specified by its locale, i.e., its neighbors. This, and similar findings prompted Lawrence and Levine to state: "It is time to move on and donate mosaic and regulative development to the archives" [4]. However, the fact remains that if one defines regulated and mosaic in terms of the first and second cleavage, regulated embryos do exist, and they explain the occurrence of identical twins.

## Cancer theories and their impact on the comprehensibility of the disease

Cancer research has been undergoing a comparable type of epistemological crisis for the last forty years, a far too long stretch given the societal impact of the disease. Until Theodor Boveri's proposal of the precursor of the somatic mutation theory in 1914, cancer was considered a tissue-based problem akin to altered embryonic development [5,6]. Boveri's proposal that cancer was caused, instead, by alterations of the genetic material shifted the cancer problem from the relational, tissue-based one, to an autonomous phenomenon occurring inside a cell. The philosopher and historian Lenny Moss identified a change of perception in the twentieth century that led to the "phylogenetic turn" whereby "... the gene and the genetic program became understood to be the principal means by which adapted form is acquired; the theater of adaptation changed from that of individual life histories, that is ontogenies, to that of populations over multiple generations, that is phylogenies" [7]. During the last decades of the 20^th ^century until now, biologists focused on the cell's interior and the somatic mutation theory became the dominant view in carcinogenesis. From this perspective, cancer became a disease caused by mutations in the DNA of a single founder cell; the tissue-based view did not disappear completely but was mostly disregarded by the mainstream.

Until the advent of the oncogene theory in the 1970s, there were multiple competing theories to explain cancer that differed at the level of biological organization in which carcinogenesis occurred. Thus, discussions centered on whether cancer was either a problem of control of cell proliferation and/or of cell differentiation, or of tissue organization [8]. Clyde Dawe [9], J.W.Orr [10], Beatrice Mintz [11], G. Barry Pierce [12], and others offered compelling evidence for carcinogenesis to be considered as a tissue-based phenomenon akin to development gone awry. Notwithstanding, around the 1960s and 70s a narrow reductionist view based on *in vitro *transformation assays as a model for neoplasia became dominant, replacing for the most part the animal models that were used until then. Philosophers, historians, and sociologists of biology have advanced explanations about how this view achieved dominance. One of them, Joan Fujimura, proposed a sociological explanation that connected the interests of molecular biologists and the rise of the genetic engineering biotechnology industry into constructing "doable problems" such as "Are there molecular changes in the cellular proto-oncogenes of tumor cells?" [13]. Michel Morange proposed, instead, an epistemic explanation implying that the oncogene theory became attractive to researchers because it suggested a straight-forward research program. Within this narrow context, the products of oncogenes participated in signal transduction conveying messages from the extracellular milieu through membrane receptors to the nucleus and these messages regulated cell division and proliferation. However, when proto-oncogenes were found in yeast, the carcinogenesis hypotheses that favored development and differentiation as central to the neoplastic process were challenged [14]. Finally, Ton van Helvoort, suggested that the oncogene theory linked exogenous (environmental) and endogenous (genetic) explanations of cancer in a single paradigm [15].

In over 40 years of dominance by the oncogene theory, hundreds of oncogenes [16] and dozens of suppressor genes [17] have been described. It is therefore reasonable to concur with the assessment by William C. Hahn and Robert A. Weinberg that, "For those who believe in the simplification and rationalization of the cancer process, the actual course of research on the molecular basis of cancer has been largely disappointing. Rather than revealing a small number of genetic and biochemical determinants operating within cancer cells, molecular analyses of human cancers have revealed a bewilderingly complex array of such factors" [18]. This assertion is in direct contradiction with the expectations that gave rise to the concept of oncogenes, which acquired their name because of their presumed "dominant" behavior; that is, at first, only one of them was expected to achieve the expression of a "transformed" phenotype, as suggested by experiments using Rous sarcoma viruses and primary cultures of chicken cells. As inconsistencies grew, the number of oncogenes increased.

A subsequent revised version of the oncogene theory, according to Hahn and Weinberg, proposed "... that the pathogenesis of human cancers is governed by a set of genetic and biochemical rules that apply to most and perhaps all types of human tumors. We believe that the identities of the mutant genes in human tumor cells will one day be conceptualized in terms of these underlying rules." The proposal was thus, to "... outline the basic rules governing the neoplastic transformation of normal human cells". According to Hahn and Weinberg, one had to look at certain properties of cancer cells such as "... to generate their own mitogenic signals, to resist exogenous growth-inhibitory signals, to evade apoptosis, to proliferate without limits (i.e., to undergo immortalization), to acquire vasculature (i.e., to undergo angiogenesis), and in more advanced cancers, to invade and metastasize."[18] Central to this revised view as well as to the original one, dubbed as "the hallmarks of cancer" was the idea that carcinogenesis is a *cell-based *problem due to mutations that cause *the founder cancer cell and its progeny *to "proliferate without limits".

The search for unifying rules was thwarted by conflicting reports from within the oncogene paradigm. Namely, "Oncogenes and tumor suppressor genes are important not only for cell proliferation but also for cell fate determination (differentiation, senescence, and apoptosis), their effects often depending on the type of cell in which they are expressed. Thus, overexpression of a given oncogene can enhance growth in one cell type but inhibit growth or induce apoptosis in another" [19]. Hence, as stated above, when data did not fit the oncogene theory, *ad hoc *explanations were proposed [20]. These interpretations of convenience lead to a situation whereby any possible conclusion was accepted as valid, because no alternative concept was ever disproved. Rather, a new layer of complexity was added. If something did not work as expected, it was blamed on its particular context and the unfathomable complexity of cancer. In sum, something could be anything and its opposite.

Admittedly, while the oncogene theory became dominant, much was learned about the role of cell-cell, tissue-tissue, and cell-extracellular matrix interactions in morphogenesis during development [6]. Meanwhile, in the last decade, the application of the morphogenetic field concept to cancer research revealed that when carcinogen exposed stroma was recombined with normal unexposed epithelial cells the latter underwent carcinogenesis [21,22]. Conversely, a neoplastic phenotype was reversed when cells from a neoplasia were placed in a normal environment [23,24]. Thus, interactions among cells in a tissue determine normal and neoplastic behavior, and imply that the neoplastic phenotype is reversible. However, in the current pervasive "anything goes" atmosphere, the findings of the alternative tissue-based theory, namely, the tissue organization field theory, are also being incorporated into the oncogene theory, like in "... incipient neoplasias begin the interplay by recruiting and activating stromal cell types that assemble into an initial preneoplastic stroma, which in turn responds reciprocally by enhancing the neoplastic phenotypes of the nearby cancer cells [25]." Hence, for those who are committed to explain carcinogenesis through a sub-cellular (mutational) strategy, the causal role of the stroma in carcinogenesis is transformed into the problem of how mutated genes affect the interactions of the cancer cells harboring these genes with otherwise normal neighboring cells. This is another example of a premature synthesis we were referring to above.

Another example of this premature synthesis is provided by the interpretation of experiments conducted in a surrogate 3D culture model. On the one hand, the authors state that "The outgrowth of sporadic mutant cells within tightly regulated cellular environments is fundamental to tumour evolution... " while on the other, they acknowledge that mutations are not sufficient to predict neoplastic behavior. By simultaneously embracing both hypotheses (the somatic mutation theory and the tissue organization field theory), a normal phenomenon during development, i.e., the capacity of an epithelium to extrude cells, is re-interpreted within a carcinogenesis context: "... a cell translocation mechanism allows sporadic mutant cells to evade suppressive micro-environments and elicits clonal selection for survival and proliferative expansion outside the native niches of these cells." Again, if carcinogenesis is a cell-intrinsic problem where mutations in oncogenes increase proliferation, it becomes problematic to graft into this theory an inhibitory role by the neighboring tissue. Moreover, not all the oncogenes investigated had this effect, and the translocation effect seems to be related instead to cell-to-cell and cell-to-matrix adhesion and to the effect of metalloproteases [26]. In experiments also performed in a surrogate 3D culture model, it was shown once more that at the single-cell level, "a cancer cell" and a normal cell behave similarly [27], thus confirming that no alleged qualitative differences exist between a normal cell and a tumor-derived (mutated) cell. The qualitative different properties between normality and carcinogenesis become patent at the tissue-level of biological complexity. That is, single cells belonging to a tumor mass retain the fundamental qualitative behavioral properties of normal cells, i.e., proliferation and motility [6,20,28].

As noted at the beginning of this Commentary, choosing between competing premises and testing hypothesis have been central components of experimental science since its inception during the Renaissance. Only after a rigorous weeding-out process, will a synthesis among different theories be justified. One of the main reasons for the reticence of researchers to deal with contradictions within the somatic mutation theory lies in the massive cost in time and resources needed to attack them experimentally, and the uncertainty that such effort may, after all, not bring about the anticipated results. Indeed, this feared outcome may also have deleterious consequences career-wise. Thus, experimental biologists are justifiably concerned when answering the relevant questions..., how and by whom can these problems be objectively judged and addressed?

## Systems Biology to the rescue?

The shortcomings of the somatic mutation theory both to explain carcinogenesis and to be translated into an effective therapeutic strategy are now being recognized even by those who side with it [29]. The reemergence of Systems Biology offers an opportunity to overcome the impasse. At present, there are various currents among its practitioners. O'Malley and Dupre [30] call the genetic approach 'pragmatic systems biology,' which is centered around large-scale molecular interactions, such as gene networks, while the organicist approach, called 'systems-theoretic biology', is centered on system principles. The differences between both approaches are not technical but rather philosophical, given that both are committed to mathematical modeling. The former uses bottom-up reductionistic approaches, the latter uses more "organicist" approaches that take into consideration bottom-up and top-down causality [31-33]. The tools of mathematical modeling and computer simulation, guided by a sound epistemological foundation, have the potential of providing the means to address the widening intellectual vacuum both theoretically and pragmatically. We hasten to add that a "sound epistemological foundation" is central to the usefulness of the outcome. Whenever the physical, tri-dimensional nature of organisms and their organs and tissues is being considered, and the very nature of life as a process is acknowledged, we have in fact, made important decisions about which type of Systems Biology we are adopting. Mathematical modeling and computer simulation enables researchers the boldness to choose premises and temporarily reject data sets without having to commit prematurely to a program of expensive and time-consuming 'wet' experiments. This exploratory role of Systems Biology may become central to breaking the habit of fixing lacks of fit with *ad hoc *explanations instead of taking a bold and critical look at the premises that were adopted [32,34]. The generation of counterintuitive *in silico *results may inspire new 'wet' research for both model validation and hypothesis testing. In fact, this is already happening as shown by a challenge to the widely accepted "hallmarks of cancer" that assumes that cancer cells actively evade cell death [35].

Returning to Driesch and Roux, we now know that, regarding the first and second cleavages, on the one hand, vertebrate development is regulated and that, on the other, in some invertebrate species development is mosaic, but any given organism expresses only one of these two modes. In this context, cancer research should benefit from rejoining the long and successful tradition in the exact sciences and in the biology of yore of discarding premises and falsifying hypotheses. And yes, cancer research in particular and biology at large should welcome the Systems Biology approach, originally outlined by Paul Weiss and Ludwig von Bertalanffy [36,37] who envisioned the advantages of adopting an organicist approach to the understanding of the living [38,39]. A new methodological outlook where mathematicians will join biologists in having an active participation in the design, exploration and interpretation of these subjects seems now necessary and timely. Simultaneously, the merging of this complementary expertise may have the added advantage of bringing into cancer biology a tradition whereby theories "... must be genuinely testable, and therefore subject to the possibility of rejection."

## Competing interests

The authors declare that they have no competing interests.

## Authors' contributions

Both authors contributed equally to the development of the ideas and the writing of this review. Both authors read and approved the final manuscript.

## References

[B1] Ayala FJ Biology as an autonomous science Am Sci 1968 56 207 221 5684549

[B2] Creath R, Maienschein J Competing epistemologies and developmental biology Biology and Epistemology, Cambridge Studies in Philosophy and Biology 2000 Cambridge, U.K.: Cambridge University Press 122 137

[B3] Gilbert SF The embryological origins of the gene theory J Hist Biol 1978 11 307 351 10.1007/BF00389303 11610436

[B4] Lawrence PA Levine M Mosaic and regulative development: two faces of one coin Curr Biol 2006 16 R236 R239 10.1016/j.cub.2006.03.016 16581495

[B5] Triolo VA Nineteenth century foundations of cancer research advances in tumor pathology, nomenclature, and theories of oncogenesis Cancer Res 1965 25 76 98 14264062

[B6] Soto AM Sonnenschein C The tissue organization field theory of cancer: A testable replacement for the somatic mutation theory Bioessays 2011 33 332 340 10.1002/bies.201100025 21503935 PMC3933226

[B7] Moss L What Genes Can't Do 2003 Cambridge, MA: MIT Press

[B8] Smithers DW Cancer: an attack of cytologism Lancet 1962 279 493 499 10.1016/S0140-6736(62)91475-7 13914465

[B9] Dawe CJ Morgan WD Slatick MS Influence of epithelio-mesenchymal interactions of tumor induction by polyoma virus Int J Cancer 1966 1 419 450 10.1002/ijc.2910010504 4287947

[B10] Orr JW Spencer AT Tarin D Transplantation studies of the role of the stroma in epidermal carcinogenesis Tissue Interactions in Carcinogenesis 1972 London: Academic Press 291 304

[B11] Illmensee K Mintz B Totipotency and normal differentiation of single teratocarcinoma cell cloned by injection into blastocysts Proc Natl Acad Sci USA 1976 73 549 553 10.1073/pnas.73.2.549 1061157 PMC335947

[B12] Pierce GB Shikes R Fink LM Cancer: A Problem of Developmental Biology 1978 Englewoods Cliffs, NJ: Prentice-Hall

[B13] Fujimura J Crafting Science 1996 Cambridge: Harvard University Press

[B14] Morange M From the regulatory vision of cancer to the oncogene paradigm J Hist Biol 1997 30 1 29 10.1023/A:1004255309721 11618978

[B15] van Helvoort T A century of research into the cause of cancer: Is the new oncogenes paradigm revolutionary? Hist Phil Life Sci 1999 21 293 330 11197187

[B16] Proto-oncogenes to oncogenes to cancer http://www.nature.com/scitable/topicpage/proto-oncogenes-to-oncogenes-to-cancer-883

[B17] Tumor suppressor (TS) genes and the two-hit hypothesis http://www.nature.com/scitable/topicpage/tumor-suppressor-ts-genes-and-the-two-887

[B18] Hahn WC Weinberg RA Mechanisms of disease: Rules for making human tumor cells New Engl J Med 2002 347 1593 1603 10.1056/NEJMra021902 12432047

[B19] Weinstein IB Cancer. Addiction to oncogenes--the Achilles heal of cancer Science 2002 297 63 64 10.1126/science.1073096 12098689

[B20] Sonnenschein C Soto AM The death of the cancer cell Cancer Res 2011 71 4334 4337 10.1158/0008-5472.CAN-11-0639 21507929 PMC3129433

[B21] Maffini MV Soto AM Calabro JM Ucci AA Sonnenschein C The stroma as a crucial target in rat mammary gland carcinogenesis J Cell Sci 2004 117 1495 1502 10.1242/jcs.01000 14996910

[B22] Nguyen DH Oketch-Rabah HA Illa-Bochaca I Geyer FC Reis-Filho JS Mao JH Ravani SA Zavadil J Borowsky AD Jerry DJ Radiation acts on the microenvironment to affect breast carcinogenesis by distinct mechanisms that decrease cancer latency and affect tumor type Cancer Cell 2011 19 640 651 10.1016/j.ccr.2011.03.011 21575864 PMC3110779

[B23] Maffini MV Calabro JM Soto AM Sonnenschein C Stromal regulation of neoplastic development: Age-dependent normalization of neoplastic mammary cells by mammary stroma Am J Pathol 2005 167 1405 1410 10.1016/S0002-9440(10)61227-8 16251424 PMC1603788

[B24] Booth BW Boulanger CA Anderson LH Smith GH The normal mammary microenvironment suppresses the tumorigenic phenotype of mouse mammary tumor virus-neu-transformed mammary tumor cells Oncogene 2011 30 679 689 10.1038/onc.2010.439 20890308 PMC3494484

[B25] Hanahan D Weinberg RA Hallmarks of cancer: the next generation Cell 2011 144 646 674 10.1016/j.cell.2011.02.013 21376230

[B26] Leung CT Brugge JS Outgrowth of single oncogene-expressing cells from suppressive epithelial environments Nature 2012 482 410 413 10.1038/nature10826 22318515 PMC3297969

[B27] Tanner K Mori H Mroue R Bruni-Cardoso A Bissell MJ Coherent angular motion in the establishment of multicellular architecture of glandular tissues Proc Natl Acad Sci USA 2012 109 1973 1978 10.1073/pnas.1119578109 22308439 PMC3277511

[B28] Sonnenschein C Soto AM The Society of Cells: Cancer and Control of Cell Proliferation 1999 New York: Springer Verlag

[B29] Michor F Liphardt J Ferrari M Widom J What does physics have to do with cancer? Nat Rev Cancer 2011 11 657 670 10.1038/nrc3092 21850037 PMC3711102

[B30] O'Malley MA Dupre J Fundamental issues in systems biology Bioessays 2005 27 1270 1276 10.1002/bies.20323 16299766

[B31] Saetzler K Sonnenschein C Soto AM Systems biology beyond networks: generating order from disorder through self-organization Semin Cancer Biol 2011 21 165 174 10.1016/j.semcancer.2011.04.004 21569848 PMC3148307

[B32] Sonnenschein C Soto AM Why Systems Biology and Cancer? Semin Cancer Biol 2011 21 147 149 10.1016/j.semcancer.2011.05.002 21621618

[B33] Soto AM Sonnenschein C Maini PK Noble D Systems biology and cancer Prog Biophys Mol Biol 2011 106 337 339 10.1016/j.pbiomolbio.2011.07.009 21843772 PMC4021577

[B34] Baker SG Kramer BS Systems biology and cancer: Promises and perils Prog Biophys Mol Biol 2011 106 410 413 10.1016/j.pbiomolbio.2011.03.002 21419159 PMC3156977

[B35] Enderling H Hahnfeldt P Cancer stem cells in solid tumors: Is 'evading apoptosis' a hallmark of cancer? Prog Biophys Mol Biol 2011 106 391 399 10.1016/j.pbiomolbio.2011.03.007 21473880

[B36] Drack M Wolkenhauer O System approaches of Weiss and Bertalanffy and their relevance for systems biology today Semin Cancer Biol 2011 21 150 155 10.1016/j.semcancer.2011.05.001 21616150

[B37] Rosslenbroich B Outline of a concept for organismic systems biology Semin Cancer Biol 2011 21 156 164 10.1016/j.semcancer.2011.06.001 21729754

[B38] Gilbert SF Sarkar S Embracing complexity: Organicism for the 21st century Dev Dyn 2000 219 1 9 10.1002/1097-0177(2000)9999:9999<::AID-DVDY1036>3.0.CO;2-A 10974666

[B39] Soto AM Sonnenschein C Emergentism as a default: cancer as a problem of tissue organization J Biosci 2005 30 103 118 10.1007/BF02705155 15824446

